# Personal

**Published:** 1891-05

**Authors:** 


### Personal.

Dr. L. S. McMurtry, of Louisville, Ky., President of the Southern Surgical and Gynecological Association, delivered two lectures before the Alumni Association of the College of Physicians and Surgeons, of Baltimore, at the College Building, corner Calvert and Saratoga streets, on Saturday evening, April 11th, at eight o'clock, and on Monday afternoon, April 13th, at five o'clock. Subject: The Pathology, Diagnosis, and Treatment of Ectopic Gestation.—*Times and Register.*

Dr. C. C. Frederick, of Buffalo, Adjunct Professor of Obstetrics in Niagara University Medical College, sailed for Europe, April 30, 1891, in the Hamburg-American steamer Augusta-Victoria. Dr. Frederick expects to be absent about six months, and will spend most of this time in study in the hospitals and clinics. He was accompanied by Mrs. Frederick.

Dr. Nicholas Senn, of Milwaukee, was promptly chosen to the chair of surgery in Rush Medical College, Chicago, made vacant by the death of Professor Parkes. Dr. Senn's acceptance was announced in the *Milwaukee Sentinel*, of April 6, 1891.

